# A Lay-User Assessment of Hepatitis C Virus Self-Testing Device Usability and Interpretation in Johannesburg, South Africa

**DOI:** 10.3390/diagnostics11030463

**Published:** 2021-03-07

**Authors:** Mohammed Majam, Alex Fischer, Elena Ivanova Reipold, Naleni Rhagnath, Vanessa Msolomba, Samanta T. Lalla-Edward

**Affiliations:** 1 Ezintsha, Faculty of Health Sciences, University of the Witwatersrand, Johannesburg 2193, South Africa; mmajam@ezintsha.org (M.M.); fischermHealth@gmail.com (A.F.); nrhagnath@ezintsha.org (N.R.); vmsolomba@ezintsha.org (V.M.); 2 Foundation for Innovative New Diagnostics, 1202 Geneva, Switzerland; elena.ivanova@finddx.org

**Keywords:** HCV, rapid diagnostic test, viral hepatitis

## Abstract

Only 20% of people with hepatitis C virus (HCV) know their status. In low-income countries diagnosis is under 10%. Self-testing for HCV antibodies (HCVST) could expand the coverage of HCV testing services. Currently, there are no stringent regulatory authority (SRA) approved HCVSTs, therefore lay-user usability of three prototype kits was assessed. This was a cross-sectional observational study conducted with 171 (CareStart n = 60, Bioline n = 52, First Response n = 59) participants. Participants were given one of the three HCVST kits with only instructions for use (IFU) and asked to perform the test in front of a professional trained in rapid diagnostic tests (RDT). Usability indices were calculated based on the correctness of performing each step of the product-specific process followed by contrived results interpretation and a post-test interview. The usability index was 93.9% for CareStart, 90.7% for Bioline and 94.9% for First Response. Most errors were on incorrect handwashing, sample collection and transfer to the test device. An average of 93.1% of contrived results were correctly interpreted, with most errors related to interpreting invalid results. Most participants (n = 167) stated they would visit a clinic after a positive result. With negative results, nearly half (28/60 (46.7%)) stated they should condomize, while just over two-thirds of participants that used Bioline (35/52 (67.3%)) and First Response (38/59 (64.4%)) said they should re-test. Most participants (n = 162) found the devices easy to use. Participants liked that self-testing was fast, private and convenient, however there were some confusion with IFU steps and pictures, finger-pricking with the lancet, collecting blood after the finger-prick, and transferring the sample/buffer. Prototype HCVST kits exhibit high usability and result interpretation by lay-users, and should be considered for SRA approval.

## 1. Introduction

Liver inflammation is referred to as hepatitis, and it is commonly caused by viral infections. Viral hepatitis has been on the rise since the 1990s and accounts for 1.4 million global deaths per year, comparable to tuberculosis (1.41 million deaths per year) and much higher than HIV (770,000 deaths per year) or malaria (405,000 deaths per year) [1,2,3,4]. Hepatitis B and C contributes the majority of chronic viral hepatitis infections and mortalities.

In 2015, an estimated 71 million people were living with hepatitis C virus (HCV) disease, and direct percutaneous exposure with infected blood led to 1.75 million new infections annually, often via the sharing of infected needles for injection-drug use and unsafe healthcare procedures [5]. In 2016, the World Health Assembly approved a global strategy to eliminate viral hepatitis as a public health threat, with the specific targets of reducing new infections by 90% and reducing deaths by 65%, by 2030 [1,5]. There is currently no vaccine for HCV, however, direct-acting antivirals introduced in 2014 have transformed the treatment options for people living with HCV [6]. An 8–12-week course of oral direct-acting antivirals has been shown to cure more than 90% of treated individuals [7,8].

The World Health Organisation (WHO) recommends treatment for all people living with HCV, however only 20% of people living with HCV globally know their status, and in low-income countries the diagnosis rate is less than 10% [5,7]. An economic analysis from Mongolia, Egypt and China suggests that a population-based test and treat approach may be cost-effective [1], and the WHO now recommends focused screening in highly affected populations and in the general population in settings with high HCV prevalence [9].

While screening can be performed using simple and affordable rapid diagnostic tests (RDT), access to HCV testing services remains a major barrier to the elimination of the disease, especially among hard-to-reach rural populations and high risk populations such as men who have sex with men and people who inject drugs [9,10]. New strategic approaches to increase HCV testing coverage are urgently needed to reach the targets of the global strategy to eliminate viral hepatitis as a public health threat by 2030 [9].

Recently, HIV self-testing (HIVST) has been identified as a facilitator to closing the testing gap for groups at high risk of HIV [11,12,13]. Self-testing is an innovative screening strategy in which people can collect their own specimen, perform a test themselves, and then interpret the result independent of traditional healthcare facilities [9]. This reduces barriers to traditional testing by increasing convenience and privacy, whilst also decreasing travel and wait times [14,15,16].

The first HIVST kit was approved for use in 2012 by the US FDA [17], and since then a strong body of evidence showing their acceptability and usability as well as increased uptake of HIV testing though HIVST [18,19,20,21,22,23,24] has led to WHO’s recommendation to use HIVST as an additional approach [16,25]. Since 2016, 40 countries have adopted national HIVST policies [26]. Based on extensive evidence available for HIVST, it is highly likely that self-testing for HCV antibodies (HCVST) has a potential to similarly increase the uptake of HCV testing. While regulators set out comprehensive requirements for self-test evaluations, including manufacturers needing to submit usability and performance data for prequalification or CE-marking, data and experience regarding HCVST remain limited in many parts of the world [27,28,29].

There is no stringent regulatory authority (SRA) approved HCVSTs, however there are some high-performance and SRA approved HCV RDTs for professional use, which may be adopted for self-testing [27,30]. Two studies evaluated the performance of oral fluid HCV RDT in the hands of untrained users and showed high agreement between the results of self-administered and research-administered RDTs [31,32], however, only one study has been published on the acceptability of HCVST. This 22-person study showed prospective acceptability towards self-testing among persons who inject drugs (PWID) in the UK [33]. Available evidence on usability, performance and acceptability of self-administered HCV RDT is very limited and there is a need for a series of evaluation studies investigating the acceptability and usability of HCVST kits in a range of HCV-prevalent settings, to guide further scale-up [27]. Evaluations conducted by the HIV Self-Testing Assessments and Research (HSTAR) programme in South Africa included usability assessments [34]. The protocol for the usability assessment of HIVST was adapted to investigate the usability of three candidate blood-based HCVST kits by lay-users, to guide manufacturers in the development of HCVST kits as they prepare for evaluations for WHO prequalification.

## 2. Materials and Methods

### 2.1. Study Design

This usability assessment was an observational cross-sectional study implemented from October 2019 to January 2020. Convenience sampling was used to recruit lay users from the general population of inner-city Johannesburg, South Africa. This evaluation of three prototype HCVST kits was directed by the protocols on self-testing which were developed based on a previously conducted usability assessment of HIVSTs in Johannesburg [35].

For each kit, a diverse sample size of at least 52 participants (170 in total) was recruited. Each of the HCVST kits were evaluated independently and in a systematic manner. Fingerprint scanning with a biometric enrolment system was used to ensure that participants were only enrolled for one device. Participants were included if they could provide written informed consent, were able to speak/read English, were 18 years of age or older, had no previous experience with self-testing, and were willing to provide fingerstick blood samples. People were excluded if they were practicing healthcare workers with prior experience using RDTs or had any condition (such as tremors, poor vision, or intoxication) that may have interfered with any of the study processes.

### 2.2. HCVST Kits

At the time of this evaluation, each of the HCVST kits were late-stage prototypes, packaged as intended for sale or distribution in South Africa. Each HCVST package included the manufacturer’s instructions for use (IFU) and all kit components required for the self-test. Three HCVST fingerstick devices were assessed: CareStart EZ HCV (CareStart) (AccessBio, Inc., Somerset, NJ, USA), SD Bioline HCV (Bioline) (Abbott Rapid Diagnostics (Pty) Ltd., Jena, Germany) and First Response HCV Card Test (First Response) (Premier Medical Corporation Pvt Ltd., Gujarat, India). All three HCVST kits were single-use tests that are considered screening tests (i.e., all positive results would require a confirmatory HCV ribonucleic acid test).

### 2.3. Data Collection

After participants provided written informed consent, a study staff accompanied them to a clinical research room to simulate a private setting for conducting the HCVST. Participants were given a packaged and sealed HCVST kit, with no familiarization or demonstration, then asked to complete the self-test while observed by a healthcare worker trained in RDT use (observer). The observer monitored the testing procedure and documented the successes and errors of each participant using a product-specific semi-structured questionnaire. The observer provided no feedback or assistance during the test. After the self-test was completed, the HCVST kits were exchanged for devices that displayed contrived results for the participant to interpret. Participants did not interpret their own HCVST, as this study did not evaluate the performance of HCVSTs.

Usability is a component of WHO prequalification therefore there have been a variety of usability studies for HIVSTs, based on the WHO prequalification guidelines for HIVST [18,19,20,21,22,23,24,32]. There is no standardized validated questionnaire for conducting HCVST usability assessments, however a recent study in South Africa used product-specific semi-structured questionnaires to conduct usability assessments on 7 HIVSTs with lay-users in the inner-city of Johannesburg [35]. Similar to this study, we have evaluated usability with a 3-part questionnaire that includes a usability index (based on a HCVST process checklist), the interpretation of contrived results and a post-test survey.

#### 2.3.1. Usability Index

For each HCVST, the usability index was calculated with a product-specific yes/no checklist that corresponded to all of the HCVST steps as outlined in each device’s IFU. The successful steps were presented as a frequency and percentage, and average of all successful steps was used to calculate the total usability index for each HCVST [35]. Some steps, such as *Did the study participant have difficulty with twisting the sterile tab?* had a negative inflection, so the ‘No’ response was used in the final usability index calculation. The final usability indices were presented as a percentage, from 0.0% (not usable) to 100.0% (very usable), with scores above 85% considered excellent [35,36,37]

#### 2.3.2. Interpreting Contrived Results

Non-functional devices had been modified by the manufacturer to display the possible outcomes from their HCVST device and these contrived results were used to evaluate each participants’ ability to correctly interpret each of the devices’ possible results: (1) non-reactive/negative; (2) reactive/positive; (3) weak reactive/weak positive (Bioline and First Response only); (4) invalid (no control line) and (5) invalid (T-line). Trained study staff observed the interpretation of the contrived results and documented all of the correctly interpreted results.

All of the participants’ HCVST devices were discarded right after the self-testing procedure and before the HCVST results appeared. Participants were not informed of their HCVST results, nor were these results interpreted or recorded anywhere. Participants that wanted to formally test for HCV were referred to the closest healthcare facilities where testing was available.

#### 2.3.3. Post-Test Survey

After completing the HCVST and interpreting the contrived results, participants were asked what they should do following positive, negative or inconclusive results, as IFUs provided recommendations for each of the possible results. Participants were also asked about their perceptions of the HCVST and the testing experience, including their most favourite and least favourite aspects of the HCVST.

### 2.4. Data Analysis

For each HCVST, data from the product-specific semi-structured questionnaires were transferred to an Excel database by the research team. Data were cleaned and coded in Excel (Microsoft Corp., Redmond, WA, USA) then quantitative data was analysed with descriptive statistics.

## 3. Results

### 3.1. Demographics

The participant demographics are presented in Table 1. The majority of participants for each device were South African (CareStart: 57/60 (95.0%); Bioline: 48/52 (92.3%); First Response: 57/59 (96.6%)), and just over half of the participants for each assessment were male (CareStart: 33/60 (55.0%); Bioline: 30/52 (57.7%); First Response: 30/59 (50.8%)). Participant age ranges varied slightly between assessments: CareStart assessment had 12/60 (20.0%) below 25 years of age, 32/60 (53.3%) participants between 26 and 35 years old, and 16/60 (26.7%) above 35 years; Bioline had 19/52 (36.5%) below 25 years of age, 18/52 (34.6%) participants between 26 and 35 years old, and 15/52 (28.8%) over 35 years old; and First Response had 26/59 (44.1%) below 25 years of age, 20/52 (33.9%) participants between 26 and 35 years old, and 13/59 (22.0%) over 35 years old. For each assessment, the most common education level was secondary education (CareStart: 25/60 (41.7%); Bioline: 25/52 (53.9%); First Response: 23/59 (39.0%)) and the majority of participants were unemployed (CareStart: 35/60 (58.3%); Bioline: 32/52 (61.5%); First Response: 44/59 (74.6%)).

### 3.2. Usability Index

The usability index for each HCVST is fully described below and in Table 2. Direct comparisons between the different HCVSTs should not be made, as each usability checklist applied product-specific steps to calculate each usability index.

#### 3.2.1. CareStart

The overall usability index of CareStart was 93.9%. All participants read and used the information sheet, while 56/60 (93.3%) selected a finger and firmly massaged it for 5–10 s and 51/60 (85.0%) cleaned the fingertip with the alcohol swab then let it air dry. None of the participants had difficulty removing the test content from the foil pouch, however one participant had difficulty twisting the sterile tab and another one had difficulty pulling the sterile Table All participants pushed firmly against the side of the finger, 58/60 (96.7%) participants squeezed behind the sample site to form a blood droplet and 48/60 (80.0%) managed to fill the tube with the correct volume of blood. Most participants (56/60 (93.3%)) placed the device on a flat surface and rotated the arm until it clicked to release the blood, 50/60 (83.3%) made sure that all of the blood was on the test strip and 59/60 (98.3%) participants used their thumb to push the button down firmly until it clicked to release the buffer.

#### 3.2.2. Bioline

For Bioline, the overall usability index was 90.7%. All participants (52/52 (100.0%)) read and used the information sheet, none (0/52 (0.0%)) had difficulty removing the test content from the foil pouch and all participants (52/52 (100.0%)) successfully placed the kit materials on a flat surface, then opened all pouches and cap. Most (45/52 (86.5%)) participants washed their hands in warm water and dried them, then 51/52 (98.1%) participants correctly chose the ring or middle finger, massaged and warmed their hands, and cleaned the finger with an alcohol swab then let it dry. Most (45/52 (86.5%)) participants successfully pressed down firmly to prick their skin, 35/52 (67.3%) participants wiped away the first drop of blood, then rubbed to create a second large drop of blood, and 43/52 (82.7%) participants used the specimen dropper to collect the drop of blood. Forty-four out of fifty-two (84.6%) participants successfully dispensed the whole blood into the round specimen well of the device, 46/52 (88.5%) participants successfully applied the plaster and 46/52 (88.5%) participants were able to twist and pull the cap to open the assay diluent, then dispense all the assay diluent tube into the square well of the device.

#### 3.2.3. First Response

The overall usability index was 94.9%. All participants (59/59 (100.0%)) read and used the information sheet and 49/59 (83.0%) participants washed their hands in warm water and soap then dried and checked the expiry date of the kit. Only 5/59 (8.5%) participants had difficulty removing the test content from the foil pouch, and 54/59 (91.5%) participants wiped their fingertip with an alcohol swab and allowed it to dry. All (59/59 (100.0%)) participants successfully removed the clear protective cap of the sterile safety lancet, successfully pressed down firmly to prick their skin, and gently squeezed their fingertip to obtain a large drop of blood. Most (51/59 (86.4%)) successfully wiped away the first drop of blood with the dry swab, 56/59 (94.9%) gently squeezed their fingertip to obtain a large second drop of blood and all (59/59 (100.0%)) participants used the specimen dropper to collect the drop of blood. Majority (53/59 (89.9%)) successfully dispensed the whole blood into the round specimen well of the device, all (59/59 (100.0%)) participants were able to twist and pull the cap to open the assay buffer. Lastly, almost all (57/59 (96.6%)) successfully added 2 drops of assay buffer into the specimen well.

### 3.3. Interpreting Contrived Results

#### 3.3.1. CareStart

For CareStart, 90.0% of the contrived results were correctly interpreted by participants. Most participants correctly interpreted the non-reactive/negative results (58/60 (96.7%)), the reactive/positive results (58/60 (96.7%)) and the invalid (no control line) results (54/60 (90.0%)), however the invalid (T-line) results were correctly interpreted by fewer participants (46/60 (76.7%)). There were no weak reactive/weak positive contrived results available to interpret.

#### 3.3.2. Bioline

Here 95.8% of the contrived results were correctly interpreted by participants. All participants (52/52 (100.0%)) correctly interpreted the reactive/positive results and the invalid (no control line) results, 49/52 (94.2%) correctly interpreted the non-reactive/negative results, 50/52 (96.2%) correctly interpreted the weak reactive/weak positive results and 46/52 (88.5%) correctly interpreted the invalid (T-line) results.

#### 3.3.3. First Response

For First Response, 97.3% of the contrived results were correctly interpreted by participants. All participants (59/59 (100.0%)) correctly interpreted the non-reactive/negative results and the reactive/positive results, while 58/59 (98.3%) correctly interpreted the weak reactive/weak positive and invalid (no control line) results, and 53/59 (89.8%) correctly interpreted the invalid (T-line) results.

These results are presented in Table 3.

### 3.4. Participant Knowledge of Processes Following an HCVST

#### 3.4.1. CareStart

When CareStart participants were asked what to do after a negative HCVST result, 28/60 (46.7%) participants would condomize, while 8/60 (13.3%) did not know what to do and none said that they would retest at all (self-test or professional). Almost all (58/60 (96.7%)) participants said they would visit a clinic after a positive HCVST result, and if the test was invalid or did not work, 19/60 (31.7%) said they would visit a clinic, while 38/60 (63.3%) said they would retest.

#### 3.4.2. Bioline

Bioline participants, when asked what to do after a negative HCVST result, 35/52 (67.3%) participants would retest, while 0/52 (0.0%) did not know what to do and none said that they would condomize. All (52/52 (100.0%)) participants stated that they would visit a clinic after a positive HCVST, and if the test was invalid or did not work, 24/52 (46.2%) said they would visit a clinic, while 28/52 (53.8%) said they would retest.

#### 3.4.3. First Response

Of the First Response participants who were asked what to do after a negative HCVST result, 38/59 (64.4%) participants would retest, while all knew what to do and 4/59 (6.8%) said that they would condomize. Almost all (57/59 (96.6%)) participants stated that they would visit a clinic after a positive HCVST, and if the test was invalid or did not work, 13/59 (23.7%) would visit a clinic, while 45/59 (76.3%) said they would retest.

### 3.5. Participant Perspectives of the HCVST Process

#### 3.5.1. CareStart

All participants used the IFU, and most participants (58/60 (96.7%)) found them easy to follow. Most participants also found the device easy to use (58/60 (96.7%)) and were confident performing it on their own (59/60 (98.3%)). Almost everyone (59/60 (98.3%)) correctly identified 15 min as the amount of time you should wait before reading the results, and 56/59 (93.3%) participants found it easy to interpret the mock results. When asked about future testing, 50/60 (83.3%) participants said they would use this test again, 52/60 (86.7%) said they would prefer to use this HCVST at home instead of going to a clinic and 55/60 (91.7%) said they would recommend this test to someone else.

When asked about the advantages of HCVST, 52/60 (86.7%) participants liked that it was easy to use, 26/60 (43.3%) liked that it was fast, 29/60 (48.3%) liked that it was private and 10/60 (16.7%)) liked that it was convenient. Most participants (52/60 (86.7%)) had no complaints about the testing process, however 4/60 (6.7%) had difficulties pricking themselves with the lancet, 1/60 (2.7%) was confused about the IFU steps and pictures and 2/60 (3.3%) did not like the lack of information on what to do after the test.

#### 3.5.2. Bioline

Almost all (51/52 (98.1%)) participants used the IFU, and 47/52 (90.4%) found it easy to follow. For the device, 45/52 (86.5%) participants found it easy to use and 46/52 (88.5%) participants were confident performing the test on their own. Almost everyone (51/52 (98.1%)) correctly identified the 15-min wait before reading the results and 48/52 (92.3%) found the contrived results easy to interpret. For future testing, 51/52 (98.1%) said they would use this test again, 42/52 (80.8%) said they would prefer to use the HCVST at home instead of going to a clinic and 51/52 (98.1%) said they would recommend this test to a sexual partner or friend.

When asked about advantages of HCVST, 44/52 (84.6%) liked that it was easy to use, 25/52 (48.1%) liked that it was fast and 5/52 (9.6%) participants liked that it was both private and convenient. Nearly two-thirds (33/52 (63.5%)) of the participants had no complaints about the HCVST process, however 14/52 (26.9%) had difficulties pricking themselves with the lancet, 2/52 (3.8%) had difficulties transferring the specimen to the buffer and 3/52 (5.8%) were confused about the IFU steps and pictures.

#### 3.5.3. First Response

All participants (59/59 (100.0%)) used the IFU, found the instructions easy to follow, and the device easy to use, while 56/59 (94.9%) participants were confident performing the test on their own. Most participants (58/59 (98.3%)) correctly identified the 15-min wait before reading the results and 55/58 (93.2%) found the mock results easy to interpret. When asked about future testing, 55/58 (93.2%) said they would use this test again, 49/59 (83.1%) said they would prefer to use this HCVST at home instead of testing at a clinic and 56/59 (94.9%) participants would recommend this test to a sexual partner or friend.

When asked about advantages of the HCVST process, 54/59 (91.5%) liked that it was easy to use, 32/59 (54.2%) liked that it was fast, 33/59 (55.9%) liked that it was private and 4/59 (6.8%) liked the convenience. Most participants (49/59 (83.1%)) had no complaints about the HCVST, however 6/59 (10.2%) had difficulties transferring the specimen to the buffer, 3/59 (5.1%) were confused about the IFU steps and pictures and 1/59 (1.7%) experienced issues with the lancet.

## 4. Discussion

This is the first study evaluating the usability of prototype blood based HCVST kits. At the time of the study, two evaluations of self-administered professional use oral fluid HCV RDTs [31,32] and one qualitative study on the acceptability had been published [33], however no reports had been published on HCVST usability to provide context for our findings. There is, however, a strong evidence base for the usability of HIVSTs, and their evaluations followed a very similar methodology. In early 2020, the HSTAR programme published an evaluation of 7 HIVSTs in the same region of Johannesburg, including 5 blood based HIVSTs [35]. These blood based HIVSTs had an average usability index of 93.6%, similar to the average usability index of the HCVST kits, which was 93.1%.

The high usability indices of the three prototype HCVST kits were supported by 86.5–100.0% of the participants that self-reported the tests were easy to use, which was consistent with the 96.3% of blood-based HIVST users from Johannesburg that also found their tests easy to use [35]. These results were also similar to other HIVST usability evaluations, including the 91% of Exacto HIVST users from the Central African Republic and the 94.4% of INSTI HIVST users in Kenya, who reported their respective HIVSTs were easy to use [38,39].

While participants reported that ease of use was the most important advantage of self-testing, participants from each prototype HCVST group liked that self-testing was fast, private and convenient. Similar attributes from HIVST studies have suggested that self-testing can remove barriers found in traditional clinic-based testing processes [14,15,16]. For HCV, barriers to testing include stigma and discrimination, which suggests that the privacy offered by HCVSTs may facilitate testing [9]. In South Africa specifically, a recent study among PWID identified barriers to accessing HCV treatment as fear of stigma, and long waiting periods [40], both barriers that can be facilitated by the speed, privacy and convenience offered by HCVST. HCVSTs have the potential to remove barriers linked with traditional HCV testing, so obtaining SRA approval for HCVSTs will help reach the 2030 targets [1,9,40], especially in key populations, like PWID.

Participants shared aspects of HCVST that they did not like, and these included unclear IFU steps and pictures, difficulties pricking themselves with the lancet, and difficulties transferring the specimen to the buffer. The lancet and specimen dropper/buffer difficulties were corroborated by the usability checklist, as steps concerning the collection of the specimen and the transfer of the specimen to the buffer were some of the only steps where more than 10% of participants made errors. The Johannesburg HIVST study identified difficulties collecting and transferring samples [35], as did an Atomo HIVST usability study in Cape Town, which reported lancing errors, the inability to collect a sufficient blood sample and transferring inadequate amounts of the sample [41]. Some of the HCV participants that cited difficulty with the IFU directly referenced the steps surrounding the collection and transfer of the specimen, which suggests that errors in specimen collection and transfer may be reduced by making clarifications/simplifications to the IFU.

On the HCVST checklists, almost 15% of participants did not wash and dry their hands according to the IFU, and over 10% of participants made mistakes wiping the first drop of blood away before collecting a sample. While these steps did not prevent the participant from completing the HCVST process, they may compromise the quality of the sample, possibly resulting in erroneous results. To see if errors in these steps would affect the accuracy of results and to determine the performance of HCVST, larger studies, similar to those for HIVST prequalification [23,24,25] should be conducted. While evaluation of the performance of HCVST prototypes was beyond the scope of the current study, we have assessed the ability of lay users to interpret the results of HCVST using contrived HCVST devices. For all three HCVST, contrived results were interpreted correctly by more than 90% of the participants. Invalid (T-line) results were interpreted correctly by 75–90% of the participants. The Johannesburg HIVST study did not have a contrived invalid (T-line) result, however the interpretation of contrived negative, positive, weak positive and invalid (no control) showed similarly high successful interpretation [35].

This study has shown high usability of prototype HCVST and has been useful in informing the improved development of blood based HCVST kits. While future usability studies on HCVST may lend from the methodologies described here, they should include more robust sample sizes from diverse populations and regions, especially in high-prevalence settings and populations [27]. Furthermore, additional performance evaluations of HCVST kits will be needed primarily because high performance quality assured HCVST tools are crucial to enable further implementation research and policy development.

Lockdown restrictions to halt the spread of COVID-19, combined with over-burdened healthcare systems have led to the disruption of hepatitis screening programmes, with some countries completely halting hepatitis screening programmes [42]. Predictive models have shown that a 1-year delay in hepatitis interventions could lead to over 70,000 excess deaths by 2030, compared to the baseline scenario with no delay [43]. Currently there is no WHO recommendation on HCVST. The scaling up of HIVST during the pandemic, has been suggested to combat a similar drop in facility-based HIV screening [44], and the scaling up of HCVST could also fill the testing void due to COVID-19. It is therefore important for the WHO to take this into account as they consider updated guidelines on HCVST in early 2021 [45].

### Limitations

The convenience sampling used in this study may have caused a sampling bias, as different proportions of sub-demographics were present between devices, specifically the participants’ employment status. No key populations were involved in the study, so findings may be not applicable for these populations. The devices were evaluated in series to ensure that there was no cross-contamination, but the sampling of participants from the same community may have resulted in participants who enrolled later in the study becoming more aware of HCVST towards the end of the study. Furthermore, the participants from the same community have been previously sampled to participate in studies with HIVST. Although previous experience with any self-test excluded someone from participating in this study, they may have heard about self-testing from others in the community.

Although data collection was directed by previous HIVST usability assessments, there is no standardized usability test for HCVST, and at the time of data collection, there were no published studies of HCVST to compare methodologies with. The semi-structured product-specific checklists allowed for the quantification of usability through a usability index, however each device checklist was slightly different, which prohibited direct comparisons between devices. No diagnostic results were collected, so there was no way to determine whether user errors observed during study actually led to erroneous test results.

## 5. Conclusions

This HCVST study has shown that evaluated prototype blood-based HCVST kits exhibit high usability in the hands of untrained lay-users, with respect to their usability index, contrived results interpretation, and direct user feedback. Although there are no other published usability assessments of blood-based HCVST to give context to these results, our findings are in agreement with high usability scores for HIVSTs in similar settings [35,38,39,40]. Given the high usability of these prototype HCVSTs, and their potential to remove barriers to traditional HCV testing, these findings are useful in informing the improved development of blood based HCVST kits towards SRA approval.

## Figures and Tables

**Table 1:** Participant demographics.

Demographic	CareStart n (%)	Bioline n (%)	First Response n (%)
**Total n**	60 (100.0)	52 (100.0)	59 (100.0)
**Nationality**			
South African	57 (95.0)	48 (92.3)	57 (96.6)
Zimbabwean	3 (5.0)	3 (5.8)	2 (4.3)
Other	8 (4.0)	1 (1.9)	0 (0.0)
**Sex**			
Female	27 (45.0)	22 (42.3)	29 (49.2)
Male	33 (55.0)	30 (57.7)	30 (50.8)
**Age**			
18–25 years old	12 (20.0)	19 (36.5)	26 (44.1)
26–35 years old	32 (53.3)	18 (34.6)	20 (33.9)
36–45 years old	12 (20.0)	10 (19.2)	12 (20.3)
46–55 years old	4 (6.7)	3 (5.8)	0 (0.0)
Over 55 years old	0 (0.0)	2 (3.8)	1 (1.7)
**Education Level**			
Primary	18 (30.0)	7 (13.5)	19 (32.2)
Secondary	25 (41.7)	28 (53.9)	23 (39.0)
Tertiary	17 (28.3)	17 (32.7)	17 (28.8)
**Employment Status**			
Employed	22 (36.7)	10 (19.2)	2 (3.4)
Unemployed	35 (58.3)	32 (61.5)	44 (74.6)
Student	3 (5.0)	10 (19.2)	13 (22.0)

Abbreviations: n—number; %—percentage.

**Table 2:** Usability checklists and index.

Usability Checklists	Yes n (%)	No n (%)	Usability Index (%)
**CareStart**			
1. Did the study participant read/use the information sheet?	60 (100.0)	0 (0.0)	100.0
2. Did the study participant select a finger and firmly massage for 5–10 s?	56 (93.3)	4 (6.7)	93.3
3. Did the study participant clean fingertip with alcohol swab then air dry?	51 (85.0)	9 (15.0)	85.0
4. Was it difficult for the study participant to remove the test content from the foil pouch?	0 (0.0)	60 (100.0)	100
5. Did the study participant have difficulty with twisting the sterile tab?	1 (1.7)	59 (98.3)	98.3
6. Did the study participant have difficulty with pulling the sterile tab?	1 (1.7)	59 (98.3)	98.3
7. Was the study participant able to push firmly against the side of the finger?	60 (100.0)	0 (0.0)	100.0
8. Did the study participant squeeze behind the sample site to form a blood droplet?	58 (96.7)	2 (3.3)	96.7
9. Did the study participant manage to fill the tube with the correct volume of blood?	48 (80.0)	12 (20.0)	80
10. Did the study participant place the device on a flat surface and rotate the arm until it clicks to release the blood?	56 (93.3)	4 (6.7)	93.3
11. Did the study participant make sure that all blood is on the test strip?	50 (83.3)	10 (16.7)	83.3
12. Was the study participant use their thumb to push the button down firmly until it clicks to release buffer?	59 (98.3)	1 (1.7)	98.3
Total usability index			**93.9**
**Bioline**			
1. Did the study participant read/use the information sheet?	52 (100.0)	0 (0.0)	100.0
2. Was it difficult for the study participant to remove the test device from the foil pouch?	0 (0.0)	52 (100.0)	100.0
3. Did the study participant successfully place material on flat surface and open all pouches and cap?	52 (100.0)	0 (0.0)	100.0
4. Did the study participant wash hands in warm water and dry?	45 (86.5)	7 (13.5)	86.5
5. Did the study participant correctly choose the ring or middle finger?	51 (98.1)	1 (1.9)	98.1
6. Did the study participant massage and warm their hands?	51 (98.1)	1 (1.9)	98.1
7. Did the study participant clean the finger with alcohol swab and let it dry?	51 (98.1)	1 (1.9)	98.1
8. Did the study participant successfully press down firmly to prick their skin?	45 (86.5)	7 (13.5)	86.5
9. Did the study participant successfully wipe away the first drop of blood with tissue and then rub create a second large drop of blood?	35 (67.3)	17 (32.7)	67.3
10. Did the study participant use the specimen dropper to collect the drop of blood?	43 (82.7)	9 (17.3)	82.7
11. Did the study participant successfully dispense the whole blood into the round specimen well (marked S’) of the device?	44 (84.6)	8 (15.4)	84.6
12. Did the study participant successfully apply in plaster?	46 (88.5)	6 (11.5)	88.5
13. Was the study participant able to twist and pull cap to open assay diluent, then dispense all the assay diluent tube into the square well of the device?	46 (88.5)	6 (11.5)	88.5
Total usability index			**90.7**
**First Response**			
1. Did the study participant read/use the information sheet?	59 (100.0)	0 (0.0)	100.0
2. Did the study participant wash hands in warm water and soap then dry and check the expiry date of the kit?	49 (83.0)	10 (17.0)	83.0
3. Was it difficult for the study participant to remove the test device from the foil pouch?	5 (8.5)	54 (91.5)	91.5
4. Did the participant wipe fingertip using alcohol swab and allow to dry (15 s)?	54 (91.5)	5 (8.5)	91.5
5. Did the study participant successfully remove the clear protective cap of the sterile safety lancet?	59 (100.0)	0 (0.0)	100.0
6. Did the study participant successfully press down firmly to prick their skin?	59 (100.0)	0 (0.0)	100.0
7. Did the study participant gently squeeze fingertip to obtain a large drop of blood?	59 (100.0)	0 (0.0)	100.0
8. Did the study participant successfully wipe away the first drop of blood with dry swab?	51 (86.4)	8 (13.6)	86.4
9. Did the study participant gently squeeze fingertip to obtain a large second drop of blood?	56 (94.9)	3 (5.1)	94.9
10. Did the study participant use the specimen dropper to collect the drop of blood?	59 (100.0)	0 (0.0)	100.0
11. Did the study participant successfully dispense the whole blood into the round specimen well (marked ‘S’) of the device?	53 (89.8)	6 (10.2)	89.8
12. Was the study participant able to twist and pull cap to open the assay buffer?	59 (100.0)	0 (0.0)	100.0
13. Was the study participant able to add 2 drops of assay buffer into the specimen well?	57 (96.6)	2 (3.4)	96.6
Total usability index			**94.9**

Abbreviations: n—number; %—percentage; IFU—information for use.

**Table 3:** Usability outcomes and user perceptions.

**Outcomes**	**CareStart** **(n = 60)**	**Bioline (n = 52)**	**First Response** **(n = 59)**
	**n (%)**	**n (%)**	**n (%)**
**Interpreting contrived results**			
Non-reactive/Negative	58 (96.7)	49 (94.2)	59 (100.0)
Reactive/Positive	58 (96.7)	52 (100.0)	59 (100.0)
Weak reactive/Weak positive	n/a	50 (96.2)	58 (98.3)
Invalid (no control line)	54 (90.0)	52 (100.0)	58 (98.3)
Invalid (T-line)	46 (76.7)	46 (88.5)	53 (89.8)
Average	54.0 (90.0)	49.8 (95.8)	57.4 (97.3)
**What to do after HCVST**			
***If Negative/Non-Reactive***			
Do not know	8 (13.3)	0 (0.0)	0 (0.0)
Condomize	28 (46.7)	0 (0.0)	4 (6.8)
Retest	0 (0.0)	35 (67.3)	38 (64.4)
Other	24 (40.0)	17 (32.7)	17 (28.8)
***If Positive/Reactive***			
Visit clinic	58 (96.7)	52 (100.0)	57 (96.6)
Other	2 (3.3)	0 (0.0)	2 (3.4)
***If Invalid/test did not work***			
Visit clinic	19 (31.7)	24 (46.2)	13 (23.7)
Retest	38 (63.3)	28 (53.8)	45 (76.3)
Other	3 (5.0)	0 (0.0)	0 (0.0)
Participant perceptions of the HCVST process *			
1. Did you use the instructions sheet?	60 (100.0)	51 (98.1)	59 (100.0)
2. Were the instructions easy to follow?	58 (96.7)	47 (90.4)	59 (100.0)
3. Was the device easy to use?	58 (96.7)	45 (86.5)	59 (100.0)
4. Did you wait the stipulated time before reading the result?	59 (98.3)	51 (98.1)	58 (98.3)
5. Were you confident with performing this test on your own?	59 (98.3)	46 (88.5)	56 (94.9)
6. Was it easy for you to INTERPRET the mock results?	58 (98.3)	48 (92.3)	55 (93.2)
7. Would you use this test again?	50 (83.3)	51 (98.1)	55 (93.2)
8. Would you prefer to use this test at home instead of getting tested at a clinic?	52 (86.7)	42 (80.8)	49 (83.1)
9. Would you recommend this test to a sexual partner, friend?	55 (91.7)	51 (98.1)	56 (94.9)
**Favourite things about the HCVST**			
Easy to use	52 (86.7) *	44 (84.6)	54 (91.5)
Fast	26 (43.3)	25 (48.1)	32 (54.2)
Private	29 (48.3)	5 (9.6)	33 (55.9)
Convenient	10 (16.7)	5 (9.6)	4 (6.8)
Other	3 (5.0)	1 (1.9)	5 (8.5)
**Least favourite things about the HCVST**			
No complaints	52 (86.7) **	33 (63.5)	49 (83.1)
Lancet difficulties	4 (6.7)	14 (26.9)	1 (1.7)
Specimen dropper/buffer difficulties	0 (0.0)	2 (3.8)	6 (10.2)
Confusion about IFU steps and pictures	1 (1.7)	3 (5.8)	3 (5.1)
No info on post-test guidance	2 (3.3)	0 (0.0)	0 (0.0)
Other	1 (1.7)	1 (1.9)	0 (0.0)

Abbreviations: n—number; %—percentage; IFU—instructions for use. Notes: * reported values are for yes answers ** percentages may not add up to 100 as participants were able to choose multiple answers.

## Data Availability

The data presented in this study are available on request from the corresponding author. The data are not publicly available to maintain participant confidentiality.
